# Arterial stiffness and biochemical profiles in prehypertensive, normotensive, and controlled hypertensive individuals: a cross-sectional study

**DOI:** 10.3389/fcvm.2025.1640622

**Published:** 2025-12-04

**Authors:** José Fernando Vilela-Martin, Tatiana Palotta Minari, Marco A. Vieira-da-Silva, Letícia Aparecida Barufi Fernandes, Marco Aurélio de Almeida, Valquíria da Silva Lopes, Kleber Aparecido de Oliveira, Jessica Rodrigues Roma Uyemura, Heitor Moreno, Juan Carlos Yugar-Toledo, Luciana Neves Cosenso-Martin

**Affiliations:** 1 Hypertension Clinic, Internal Medicine Department, State Medical School at São José do Rio Preto (FAMERP), São José do Rio Preto, Brazil; 2 Internal Medicine Department, State University of Campinas (UNICAMP), São José do Rio Preto, Brazil

**Keywords:** prehypertension, hypertension, blood pressure, arterial stiffness, pulse wave analysis, ambulatory blood pressure monitoring

## Abstract

**Introduction:**

Prehypertension predisposes individuals to hypertension as well as increased cardiovascular morbidity and mortality. Additionally, central blood pressure and arterial stiffness indices have been linked to higher cardiovascular mortality rates. This study aimed to compare peripheral and central hemodynamic parameters—including blood pressure, pulse wave velocity, and nocturnal dipping—among normotensive, prehypertensive, and controlled hypertensive individuals, alongside the assessment of biochemical variables.

**Methods:**

The study compared clinical and biochemical evaluations and ambulatory blood pressure monitoring (ABPM) results among 47 normotensive (NT), 39 prehypertensive (PH), and 138 controlled hypertensive (CHT) individuals. Peripheral [systolic blood pressure (SBP) and diastolic blood pressure (DBP)] and central hemodynamic [central SBP (cSBP), central DBP (cDBP), and pulse wave velocity (PWV)] parameters were analyzed using ABPM. Central hemodynamic parameters were measured via brachial oscillometry with the Mobil-O-Graph® system.

**Results:**

The mean ages of NT, PH, and CHT participants were 48.3 ± 10.6, 50.1 ± 9.6, and 57.7 ± 10.9 years, respectively (*P* < 0.0001). Compared to NT, PH individuals showed higher 24-h systolic blood pressure (130.2 ± 5.6 vs. 118.7 ± 4.9 mmHg; *P* < 0.0001), central systolic pressure (125.4 ± 6.1 vs. 113.2 ± 5.3 mmHg; *P* < 0.0001), and pulse wave velocity (8.2 ± 1.1 vs. 7.6 ± 0.9 m/s; *P* = 0.01). Triglycerides levels were significantly higher in PH (178 ± 42 mg/dL) than in NT (132 ± 36 mg/dL; *P* = 0.0002) and lower than in CHT (192 ± 47 mg/dL; *P* = 0.03). Glycemia, LDL cholesterol, and total cholesterol also differed significantly between PH and CHT groups (*P* < 0.001).

**Conclusion:**

Prehypertensive individuals exhibited higher peripheral and central blood pressures compared to normotensive individuals but lower levels than controlled hypertensive patients during all three periods (24-h, wake, and sleep). These findings suggest that functional and structural alterations predisposing individuals to hypertension are already present in the prehypertensive stage.

## 1 Introduction

Prehypertension (PH) is regarded as a precursor to hypertension (HT) and is associated with increased morbidity and mortality from cardiovascular diseases (CVD), which account for approximately 32% of deaths in Brazil (1–4). The classification of prehypertension adopted in this study follows the criteria established by the Seventh Report of the Joint National Committee (JNC 7), which defined systolic blood pressure between 120 and 139 mmHg and/or diastolic pressure between 80 and 89 mmHg as prehypertensive (5).

Although this guideline has historical relevance and was widely used at the time of data collection, more recent recommendations—such as the 2017 ACC/AHA guideline and the 2020 Brazilian Hypertension Guidelines—have redefined blood pressure categories, often incorporating lower thresholds for diagnosis and intervention (3, 4). Despite these updates, the JNC 7 framework remains useful for comparative purposes and continues to be referenced in epidemiological studies (5). Based on this classification, the prevalence of prehypertension was estimated at approximately 38% in earlier reports, although more recent data suggest a modest decline in prevalence over the past decades, particularly in high-income populations (2–6).

The risk of developing CVD, which begins at BP levels of 115/75 mmHg, doubles with every 20 mmHg increase in systolic BP (SBP) and 10 mmHg increase in diastolic BP (DBP) (1–4, 6). Consequently, prehypertension has become the most prevalent risk factor for developing HT and is recognized as a significant risk factor for target organ damage, including myocardial infarction and coronary atherosclerotic disease, resulting in higher mortality (2, 7–15). Data from the Strong Heart Study (SHS) indicate that individuals within this BP category have a 1.8-fold higher risk of CVD than normotensive individuals, corresponding to an absolute increase of six cardiovascular events per 1,000 individuals per year (11–17).

Along with BP, functional and morphological parameters, such as heart rate (HR), pulse pressure (PP), mean arterial pressure (MAP), end-systolic stress, and end-isovolumetric systolic stress, have been shown to be significantly higher in prehypertension compared to normotension (13, 18–21). Other markers, including central blood pressure, augmentation index (AI75%), and pulse wave velocity (PWV), widely used as indicators of arterial stiffness, have been linked to increased cardiovascular mortality and are considered superior predictors of CVD (8, 14, 15, 22–27). Central blood pressure represents a blood pressure measured in the aortic root, and AI75% is estimated as a composite marker of wave reflections and arterial stiffness. On the other hand, PWV represents an index of elastic-type aortic stiffness. Studies also suggest that arterial stiffness correlates with age, triglycerides levels, SBP, 24-h PP, urinary albumin excretion, and carotid artery intima-media thickness (14, 15, 28–32). These factors are prevalent in the prehypertensive population, with 64% of individuals under 60% and 94% over 60 years old exhibiting one or more risk factors for CVD (6, 7, 16, 33–38).

Consequently, early diagnosis and implementation of therapeutic measures in prehypertensive individuals aim to mitigate the risk of HT, CVD, and mortality (1, 7, 16, 17). While the diagnosis of HT typically involves detecting consistently elevated BP levels in clinical settings, ambulatory blood pressure monitoring (ABPM) enables the indirect and continuous recording of BP over 24 h or more (39–42). This method facilitates the identification of circadian BP variations, the development of therapeutic and prognostic strategies, and the evaluation of antihypertensive treatment efficacy (18, 43–47).

Given the clinical relevance of arterial stiffness assessment, it is essential to consider the methodologies available for its measurement. Among these, carotid-femoral pulse wave velocity (cfPWV) is widely regarded as the gold standard due to its direct evaluation of aortic stiffness and robust predictive value for cardiovascular outcomes (14, 15, 19, 24, 25). However, cfPWV requires specialized equipment and trained personnel, which may limit its feasibility in routine clinical practice and large-scale epidemiological studies. As an alternative, oscillometric devices such as the Mobil-O-Graph® estimate central hemodynamic parameters and PWV through mathematical modeling of brachial pressure waveforms (24). These methods offer practical advantages—including ease of use, noninvasiveness, and suitability for 24-h ambulatory monitoring—but rely on indirect calculations and single-segment assessments that may not fully reflect central arterial stiffness. Consequently, interpretation of results obtained via such devices should be approached with consideration of these methodological constraints (15, 19, 24, 25).

Among the variables derived from ABPM, sleep SBP, 24-h SBP, and wake SBP have demonstrated the strongest associations with cardiovascular events and target organ damage (18, 19). In particular, nocturnal blood pressure dipping—characterized by a physiological decline in BP during sleep—has emerged as a key prognostic marker, with reduced or absent dipping linked to increased cardiovascular risk and end-organ injury (20). Building on this evidence, the present study aimed to comprehensively evaluate peripheral hemodynamic parameters (BP, MAP, HR, and PP) and central indices (cSBP, cDBP, and PWV) in prehypertensive individuals, comparing them to normotensive and controlled hypertensive subjects. This approach seeks to elucidate early vascular and metabolic alterations across distinct blood pressure categories under real-life conditions.

Therefore, the objective of this study was to compare peripheral and central hemodynamic parameters—including blood pressure, pulse wave velocity, and nocturnal dipping—as well as biochemical variables such as glycemia, lipid profile, renal function markers, and uric acid levels across normotensive, prehypertensive, and controlled hypertensive individuals. The originality of this work lies in its integration of 24-h ABPM monitoring with central arterial stiffness indices using a validated oscillometric method, allowing for real-life assessment of vascular function. Unlike previous studies that focused solely on prehypertension, this design enables a broader understanding of cardiovascular remodeling across the blood pressure spectrum, while emphasizing the prognostic relevance of nocturnal dipping—a parameter often overlooked in conventional evaluations.

## 2 Methods

### 2.1 Subjects

This study was conducted at the Hypertension Outpatient Clinic of the Medical School of São José do Rio Preto (FAMERP), in accordance with the ethical principles outlined in the Declaration of Helsinki. Ethical approval was granted by the Human Research Ethics Committee of FAMERP under protocol CAAE: 84121518.1.0000.5415 on March 2, 2018. Participant recruitment occurred between March 2018 and December 2019.

The target population comprised adult patients attending the Hypertension Outpatient Clinic for routine evaluation or follow-up. During the recruitment period, approximately 500 patients were seen at the clinic. Of these, 278 individuals were assessed for eligibility. Participants were invited consecutively during scheduled appointments and selected through convenience sampling based on predefined inclusion criteria and willingness to participate.

A total of 54 individuals were excluded due to prior diagnosis of hypertension or use of antihypertensive medication (*n* = 31), cognitive impairment (*n* = 12), pregnancy (*n* = 6), or inability to complete blood pressure measurements (*n* = 5). The final sample included 224 individuals aged 23–79 years, distributed as follows: 47 normotensive (NT), 39 prehypertensive (PH), and 138 controlled hypertensive (CHT) participants.

Classification was based on the mean of three office blood pressure measurements obtained during the study visit. Normotension was defined as systolic blood pressure (SBP) <120 mmHg and diastolic blood pressure (DBP) <80 mmHg without antihypertensive treatment. Prehypertension was defined as SBP between 120 and 139 mmHg and/or DBP between 80 and 89 mmHg in untreated individuals (5). Controlled hypertension was defined as SBP <140 mmHg and DBP <90 mmHg in patients undergoing antihypertensive therapy during outpatient follow-up.

Exclusion criteria included pregnancy, low life expectancy (e.g., cancer), prior diagnosis of hypertension or use of antihypertensive medication (applicable to NT and PH groups), cognitive impairment preventing study participation, and inability to perform blood pressure measurements.

### 2.2 Office blood pressure measurements

Office blood pressure measurements were performed specifically for the purposes of this study using the auscultatory method with a calibrated aneroid sphygmomanometer (Premium® brand, model P.A. MED). Device calibration was verified monthly by the institution's biomedical engineering team. All measurements were conducted by trained nursing staff who received standardized instruction in blood pressure assessment techniques. Prior to measurement, participants were asked to avoid caffeine, exercise, and smoking for at least 30 min and were seated at rest for 5 min in a quiet room with their back supported and feet flat on the floor. Three consecutive readings were taken at one-min intervals, and the mean of these values was used for classification (4).

### 2.3 Clinical and biochemical analysis

Data on age, gender, weight, height, body mass index [BMI = weight (kg)/height squared (m^2^)], medications, comorbidities, and diabetes mellitus status were obtained through interviews and verified via medical records. Participants were classified with diabetes if they were undergoing treatment with hypoglycemic agents or had fasting glucose levels ≥126 mg/dL on at least two separate occasions, in accordance with the diagnostic criteria established by the American Diabetes Association (ADA) (48).

For biochemical analyses, peripheral blood samples were collected following an overnight fast. Investigated biochemical parameters included uric acid and serum creatinine, assessed through kinetic colorimetric assays, and potassium, measured using a selective electrode and ion tests. Urine albumin excretion was measured by immunoradiometric assay in a morning urine sample. Glycemia, total cholesterol, high-density lipoprotein cholesterol (HDL-c), and triglycerides were analyzed using enzymatic colorimetric methods. The low-density lipoprotein cholesterol (LDL-c) fraction was calculated via the Friedewald formula (21). The estimated glomerular filtration rate (eGFR) was determined using the MDRD (Modification of Diet in Renal Disease) formula and the Cockroft-Gault equation (22, 23).

ABPM and laboratory tests—were performed on the same day or within a maximum interval of seven days to ensure consistency and minimize temporal variability in the data.

### 2.4 Ambulatory blood pressure monitoring (ABPM)

All participants underwent ABPM on a routine activity day using a cuff appropriately sized for the individual's arm and the Mobil-O-Graph® 24 h PWA Monitor (24, 25). The parameters recorded included SBP, DBP, mean arterial pressure (MAP), heart rate (HR), pulse pressure (PP), central SBP (cSBP), central DBP (cDBP), and PWV. Measurements were taken at 30-min intervals, with averages calculated for three time periods: 24-h, wakefulness, and sleep.

The Mobil-O-Graph® system estimates central blood pressure (cBP) using a transfer function algorithm applied to brachial pressure waveforms obtained via oscillometric measurements. This method reconstructs the aortic pressure wave based on the shape and timing of the peripheral pulse wave, allowing for noninvasive approximation of central systolic and diastolic pressures. The algorithm has been validated against invasive intra-arterial measurements and shown to provide reliable estimates of central hemodynamics in various populations. Its clinical applicability has been supported by comparative studies demonstrating consistency with tonometric and catheter-based techniques (24).

Nocturnal dipping was defined as a ≥10% reduction in SBP and DBP from wakefulness to sleep. Dipping was categorized as inverted (reduction <0%), absent (<10% reduction), or extreme (≥20% reduction), according to the validated Brazilian Guidelines (26).

### 2.5 Statistical analysis

The sample size was determined through an *a priori* power analysis using G*Power software (version 3.1). Based on a medium effect size (Cohen's *d* = 0.5), a significance level of *α* = 0.05, and a desired statistical power of 80%, the minimum required sample size was estimated at 159 participants. The final sample of 224 individuals exceeded this threshold, ensuring adequate power to detect statistically significant differences among the study groups.

Additionally, a *post hoc* power analysis was performed for key comparisons. For instance, the difference in 24-h SBP between prehypertensive and normotensive individuals (mean difference = 11.5 mmHg; pooled standard deviation ≈ 5.3 mmHg) yielded an estimated effect size of 2.17. With *α* = 0.05 and a combined sample size of 86 for these two groups, the calculated statistical power exceeded 99%, confirming the robustness of the study in detecting clinically relevant differences in primary outcomes.

Descriptive statistics were used for qualitative variables, with data presented as means ± standard deviation. Variable distribution was evaluated using the Shapiro–Wilk normality test. For comparisons, ANOVA was applied to normally distributed quantitative variables, the Kruskal–Wallis test for non-normally distributed quantitative variables, and Fisher's exact test for qualitative variables related to participant characteristics. A *p*-value <0.05 was considered statistically significant. All statistical analyses were performed using SPSS version 24.0 (SPSS Inc., Chicago, IL, USA).

## 3 Results

A total of 224 subjects (104 male, 46.4%) participated in the study, comprising 47 NT individuals (9 male, 19.1%), 39 PH subjects (34 male, 85.0%), and 138 CHT individuals (61 male, 43.5%). Gender, mean age, BMI, and biochemical variables, as well as comparisons between groups, are shown in Table 1.

**Table 1:** Clinical and biochemical parameters in normotensive, prehypertensive and controlled hypertensive individuals.

Variable	NT	PH	CHT	*p*-value (a x b x c)	a x b	a x c	b x c
	(*n* = 47)^a^	(*n* = 39)^b^	(*n* = 138)^c^				
Age (years)	48.3 ± 10.6	50.1 ± 9.6	57.7 ± 10.9	0.000**	NS	0.000**	0.000**
Male (%)	9 (19.1)	34 (85)	61 (43.5)	0.000**	0.000**	0.001**	0.000**
White (%)	42 (89.3)	38 (97.4)	121 (87.6)	NS	–	–	–
BMI (kg/m^2^)	27 ± 6.1	27.7 ± 4.4	29.4 ± 5.0	0.007**	NS	0.01*	NS
Smokers—*n* (%)	3 (6.3)	5 (12.8)	12 (8.6)	NS	–	–	–
Diabetes—*n* (%)	1 (2.1)	0	41 (29.7)	0.000**	NS	0.000**	0.000**
Biochemical parameters							
Glycemia (mg/dL)	97 ± 1.2	92.8 ± 9.7	119.6 ± 1.4	0.000**	NS	0.000**	0.000**
Creatinine (mg/dL)	0.75 ± 0.1	0.90 ± 0.1	0.96 ± 0.4	0.000**	0.000**	0.000**	NS
UA (mg/dL)	3.9 ± 0.9	6.1 ± 1.2	5.7 ± 2.0	0.000**	0.000**	0.000**	NS
eGFR (MDRD)	95.8 ± 20.8	91 ± 20.9	76.8 ± 20.6	0.000**	NS	0.000**	0.01*
Potassium (mEq/L)	4.3 ± 0.4	4.4 ± 0.2	4.4 ± 0.2	NS	–	–	–
TC (mg/dL)	195.4 ± 32.5	204.4 ± 26	184.6 ± 45.4	0.001*	NS	NS	0.000**
HDL-c (mg/dL)	57.7 ± 13	45 ± 6.4	47.9 ± 14.6	0.000**	0.000**	0.000**	NS
LDL-c (mg/dL)	114.9 ± 33.5	124.1 ± 27.6	106.1 ± 41.2	0.003*	NS	NS	0.002*
TG (mg/dL)	110.9 ± 40.5	169 ± 63	149.7 ± 102.5	0.000**	0.000**	0.000**	0.02*
SBP Office blood pressure (mmHg)	116.5 ± 13.9	130.2 ± 5.6	128.7 ± 6.3/	0.0001	0.0001	0.0001	0.06
DBP Office blood pressure (mmHg)	72.4 ± 10.6	84.1 ± 4.8	82.9 ± 5.1	0.0001	0.0001	0.0001	0.07
Drugs—*n* (%)							
Statins	–	–	44 (31.8)				
Diuretics	–		39 (28.2)				
ARB	–		67 (48.5)				
ACEi	–		42 (30.4)				
CCB	–		46 (33.3)				
B-blockers	–		34 (24.6)				
Antiaggregant and/or anticoagulant	–	–	19 (13.7)				

NT, normotensive; PH, prehypertensive; CHT, controlled hypertensive; BMI, body mass index; eGFR, estimated glomerular filtration rate; MDRD, modification of diet in renal disease; UA, uric acid; TC, total cholesterol; HDL-c, high-density lipoprotein; LDL-c, low-density lipoprotein; TG, triglycerides; ARB, angiotensin receptor blockers; ACEi, angiotensin converting enzyme inhibitors; CCB, calcium channel blockers; B-blockers, beta-blockers.

a, Normotensive group (NT, *n* = 47).

b, Prehypertensive group (PH, *n* = 39).

c, Controlled hypertensive group (CHT, *n* = 138).

* *p* < 0.05.

** *p* < 0.001; NS, non significant.

To support the classification and comparison between groups, mean office blood pressure values were recorded and statistically analyzed. The NT group presented an average systolic/diastolic BP of 116.5 ± 13.9/72.4 ± 10.6 mmHg, the PH group 130.2 ± 5.6/84.1 ± 4.8 mmHg, and the CHT group 128.7 ± 6.3/82.9 ± 5.1 mmHg (Table 1). These findings confirm that, although under antihypertensive treatment, the CHT group maintained BP levels within the controlled range, which may overlap with prehypertensive thresholds. Therefore, the inclusion of both office BP and ABPM values reinforces the validity of between-group comparisons.

Significant differences were observed in the PH group compared to the NT and CHT groups concerning gender (*p* = 0.000 for both) and triglycerides levels (*p* = 0.000 and *p* = 0.02, respectively). Compared to the NT group, the PH group also exhibited significant differences in creatinine levels (*p* = 0.000), uric acid (*p* = 0.000), and HDL-c (*p* = 0.000). When compared to the CHT group, the PH group differed significantly in age (*p* = 0.000), history of diabetes (*p* = 0.000), glucose levels (*p* = 0.000), eGFR (*p* = 0.01 for both MDRD and Cockroft-Gault models), total cholesterol (*p* = 0.000), and LDL-c (*p* = 0.002). Given the lack of differences between MDRD and Cockroft-Gault calculations, only MDRD results are presented (Table 1).

No significant differences were noted between groups for race, smoking status, or serum potassium levels. Although albuminuria levels increased across groups, no statistical significance was observed (Table 1).

### 3.1 ABPM comparisons

Over the 24-h period, the PH group exhibited significantly higher SBP compared to the NT group (130.2 ± 5.6 mmHg vs. 118.7 ± 4.9 mmHg; mean difference = 11.5 mmHg; *p* < 0.0001), and higher DBP (84.1 ± 4.8 mmHg vs. 76.3 ± 4.2 mmHg; mean difference = 7.8 mmHg; *p* < 0.0001). MAP was also elevated in PH (99.5 ± 4.7 mmHg) compared to NT (90.4 ± 4.3 mmHg; mean difference = 9.1 mmHg; *p* < 0.0001). Central systolic pressure (cSBP) and central diastolic pressure (cDBP) were higher in PH (125.4 ± 6.1 mmHg and 83.2 ± 4.6 mmHg, respectively) than in NT (113.2 ± 5.3 mmHg and 77.1 ± 4.1 mmHg; mean differences = 12.2 mmHg and 6.1 mmHg; *p* < 0.0001 and *p* = 0.002, respectively).

Compared to the CHT group, the PH group showed lower 24-h SBP (130.2 ± 5.6 mmHg vs. 134.1 ± 6.2 mmHg; mean difference = −3.9 mmHg; *p* = 0.02), pulse pressure (PP) (41.1 ± 5.2 mmHg vs. 44.3 ± 5.7 mmHg; mean difference = −3.2 mmHg; *p* = 0.02), central SBP (125.4 ± 6.1 mmHg vs. 129.7 ± 6.5 mmHg; mean difference = −4.3 mmHg; *p* = 0.01), and pulse wave velocity (PWV) (8.2 ± 1.1 m/s vs. 9.1 ± 1.3 m/s; mean difference = −0.9 m/s; *p* < 0.0001).

During wakefulness, similar trends were observed. The PH group had higher SBP (132.5 ± 5.8 mmHg vs. 120.3 ± 5.1 mmHg; mean difference = 12.2 mmHg; *p* < 0.0001), DBP (85.3 ± 4.9 mmHg vs. 77.2 ± 4.4 mmHg; mean difference = 8.1 mmHg; *p* < 0.0001), and MAP (100.7 ± 4.9 mmHg vs. 91.6 ± 4.5 mmHg; mean difference = 9.1 mmHg; *p* < 0.0001) compared to NT. Central pressures were also elevated in PH: cSBP (127.6 ± 6.3 mmHg vs. 115.1 ± 5.5 mmHg; mean difference = 12.5 mmHg; *p* < 0.0001) and cDBP (84.5 ± 4.7 mmHg vs. 78.3 ± 4.2 mmHg; mean difference = 6.2 mmHg; *p* < 0.0001).

During sleep, the PH group showed higher SBP (118.4 ± 5.2 mmHg vs. 108.7 ± 4.6 mmHg; mean difference = 9.7 mmHg; *p* = 0.01) and MAP (92.3 ± 4.6 mmHg vs. 84.7 ± 4.1 mmHg; mean difference = 7.6 mmHg; *p* = 0.007) compared to NT. Compared to CHT, PH had lower PP (39.2 ± 4.9 mmHg vs. 42.5 ± 5.3 mmHg; mean difference = −3.3 mmHg; *p* = 0.03), cSBP (123.1 ± 5.9 mmHg vs. 127.8 ± 6.2 mmHg; mean difference = −4.7 mmHg; *p* < 0.0001), and PWV (8.0 ± 1.0 m/s vs. 9.0 ± 1.2 m/s; mean difference = −1.0 m/s; *p* < 0.0001).

Mean 24-h ambulatory systolic and diastolic blood pressure values were 118.7 ± 4.9/76.3 ± 4.2 mmHg for the NT group, 130.2 ± 5.6/84.1 ± 4.8 mmHg for the PH group, and 134.1 ± 6.2/86.7 ± 5.1 mmHg for the CHT group. A comprehensive summary of biochemical, clinical, and hemodynamic comparisons—including office and ambulatory blood pressure measurements—is presented in Tables 1, 2.

**Table 2:** Peripheral and central hemodynamic parameters in normotensive, prehypertensive and controlled hypertensive individuals.

Period	Variable	NT	PH	CHT	*p*-value	a x b	a x c	b x c
		(*n* = 47)^a^	(*n* = 39)^b^	(*n* = 138)^c^				
24-h	SBP	109 ± 6.2	117.4 ± 7.3	123.7 ± 12.5	0.000**	0.000**	0.000**	0.02*
	DBP	67 ± 6.8	74.3 ± 7.5	76.5 ± 10.8	0.000**	0.000**	0.000**	NS
	MAP	85.8 ± 6.4	94.1 ± 6.5	98 ± 10.9	0.000**	0.000**	0.000**	NS
	HR	75.8 ± 8	73.5 ± 8.9	74.3 ± 10.4	NS	–	–	–
	PP	42.0 ± 5.7	42.7 ± 6.2	47.2 ± 9.0	0.000**	NS	0.001**	0.02*
	cSBP	100.4 ± 12	109.2 ± 6.8	114.9 ± 11.3	0.000**	0.000**	0.000**	0.01*
	cDBP	66.9 ± 11.8	75 ± 7.2	78 ± 11.1	0.000**	0.002*	0.000**	NS
	PWV	6.7 ± 1.24	7.0 ± 1.11	8.2 ± 1.46	0.000**	NS	0.000**	0.000**
Wakefulness	SBP	121.3 ± 8.1	125.0 ± 6.5	125.9 ± 12.6	0.000**	0.000**	0.000**	NS
	DBP	70.4 ± 6.9	78.8 ± 7.8	79.0 ± 11.3	0.000**	0.000**	0.000**	NS
	MAP	89.6 ± 5.9	98.3 ± 7.1	100.6 ± 11.0	0.000**	0.000**	0.000**	NS
	HR	79.7 ± 9.0	78.2 ± 9.1	77.9 ± 11.1	NS	–	–	–
	PP	42.0 ± 6.3	42.7 ± 6.6	46.8 ± 9.0	0.001*	NS	0.003*	0.03*
	cSBP	104.1 ± 5.7	112.3 ± 8.0	116 ± 11.5	0.000**	0.000**	0.000**	NS
	cDBP	72.3 ± 7.5	80.3 ± 7.9	81.0 ± 11.5	0.000**	0.000**	0.000**	NS
	PWV	6.8 ± 1.24	7.1 ± 1.12	8.3 ± 1.37	0.000**	NS	0.000**	0.000**
Sleep	SBP	103.1 ± 8.0	110.7 ± 8.1	119.9 ± 13.7	0.000**	0.01*	0.000**	0.000**
	DBP	61.3 ± 8.1	67.5 ± 8.2	72 ± 11.3	0.000**	0.01*	0.000**	NS
	MAP	80.6 ± 7.4	87.3 ± 7.3	93.9 ± 11.5	0.000**	0.007*	0.000**	0.006*
	HR	69.1 ± 7.7	66.2 ± 9.6	68.3 ± 9.9	NS	–	–	–
	PP	41.8 ± 5.8	43.0 ± 6.5	47.7 ± 9.4	0.000**	NS	0.001*	0.03*
	cSBP	98.6 ± 8.3	104.2 ± 7.8	113.0 ± 12.7	0.000**	NS	0.000**	0.000**
	cDBP	62.1 ± 8.0	68.3 ± 8.0	73.1 ± 11.6	0.000**	0.01*	0.000**	NS
	PWV	6.5 ± 1.26	6.8 ± 1.11	8.17 ± 1.48	0.000**	NS	0.000**	0.000**

NT, normotensive; PH, prehypertensive; CHT, controlled hypertensive; SBP, systolic blood pressure; DBP, diastolic blood pressure; MAP, mean arterial pressure; HR, heart rate; PP, pulse pressure; cSBP, central systolic blood pressure; cDBP, central diastolic blood pressure; CO, cardiac output; PVR, total vascular resistance; PWV, pulse wave velocity.

a, Normotensive group (NT, *n* = 47).

b, Prehypertensive group (PH, *n* = 39).

c, Controlled hypertensive group (CHT, *n* = 138).

* *p* < 0.05.

** *p* < 0.001; NS, non significant.

### 3.2 Nocturnal dipping

For nocturnal SBP dipping, a significant difference was observed between the PH and CHT groups (*p* = 0.003) and between the NT and CHT groups (*p* = 0.005), but not between the NT and PH groups. For diastolic nocturnal dipping, a significant difference was found only between the PH and CHT groups (*p* = 0.0006). These findings are summarized in Table 3.

**Table 3:** Nocturnal dipping in normotensive, prehypertensive and controlled hypertensive individuals.

Variable	NT	PH	CHT	a x b	a x c	b x c
	(*n* = 47)^a^	(*n* = 39)^b^	(*n* = 138)^c^			
Non-Dipping Systolic (%)	29 (61.7%)	25 (64.1%)	113 (81.8%)	NS	0.005**	0.003**
Non-Dipping Diastolic (%)	23 (48.9%)	12 (30.7%)	84 (60.8%)	NS	0.05	0.0006**

NT, normotensive; PH, prehypertensive; CHT, controlled hypertensive.

a, Normotensive group (NT, *n* = 47).

b, Prehypertensive group (PH, *n* = 39).

c, Controlled hypertensive group (CHT, *n* = 138).

*p < 0.05.

** *p* < 0.001; NS, non significant.

## 4 Discussion

This study revealed biochemical-metabolic differences among the groups in terms of glycemia, creatinine, uric acid, eGFR, total cholesterol, HDL-c, LDL-c, and triglycerides. Through ABPM, differences were observed in all three periods (24-hour, wakefulness, and sleep) for both peripheral (SBP, DBP, and MAP) and central parameters (cSBP, cDBP, and PWV) among the groups.

The observed differences in gender proportions among NT, PH, and CHT groups are consistent with prior findings, often attributed to biological and behavioral factors (27). Studies have shown that men typically present higher BP levels than premenopausal women; however, after menopause, BP increases in women, diminishing or nullifying this difference (28, 29). Hormone replacement therapy has not demonstrated significant reductions in BP for most postmenopausal women, suggesting that estrogen loss alone does not fully explain elevated BP in this population (28, 29).

The inclusion of the CHT group, despite ongoing antihypertensive therapy, was intended to provide a broader clinical perspective on the progression of vascular changes across different blood pressure categories. This comparison enables the assessment of whether early alterations in arterial stiffness and central hemodynamics observed in PH individuals resemble those found in treated hypertensive patients. Pharmacological treatment may influence biochemical and hemodynamic parameters in the CHT group—particularly lipid profiles due to statin use and PWV values due to age and blood pressure levels. Nevertheless, contrasting untreated PH with clinically controlled hypertension offers relevant insight into the continuum of cardiovascular remodeling and the potential impact of therapeutic interventions.

Regarding biochemical parameters, uric acid, creatinine, and HDL-c levels were significantly different in prehypertensive individuals compared to normotensive individuals, while glycemia, total cholesterol, LDL-c, and eGFR levels differed when comparing PH to CHT individuals. Triglycerides levels were distinct between the PH group and both the NT and CHT groups. Elevated uric acid levels, a byproduct of purine metabolism, have been associated with increased prehypertension risk (30–33), a finding found in the PH group compared to the NT group. Our study aligns with studies of Liu et al. (32, 33). The first reported a positive correlation between uric acid levels and PH incidence in a 6-year prospective cohort study (32), while the second showed that elevated levels of uric acid may be associated with increased risk of PH in the meta-analysis of 17 observational studies of approximately 79,358 participants (33). The authors demonstrated that the risk of PH increased 46% for highest levels compared with lowest levels of uric acid (33). Moreover, PH and high uric acid levels significantly increase the risk of arterial stiffness (34).

Prehypertensive individuals also exhibited higher levels of triglycerides, total cholesterol, and LDL-c, and lower HDL-c compared to both NT and CHT groups. While this finding aligns with previous research regarding normotensive individuals (35), the differences relative to the CHT group may be attributable to statin use. Statins effectively reduce cholesterol and LDL-c levels, lower CVD risk, stabilize atherosclerotic plaques, and possess anti-inflammatory properties, thus reducing cardiovascular complications and mortality (36, 37). Anyway, Vucak et al. found association between hyperuricemia and prehypertension in individuals with elevated BMI and triglycerides, suggesting an interplay between metabolic factors and PH (38), fact that corroborates with our results.

Renal function, evaluated via MDRD and Cockcroft-Gault equations, showed statistical differences between PH and CHT groups. This aligns with studies highlighting the close relationship between HT and kidney disease (39, 40). Notably, although not statistically significant, eGFR was lower in PH compared to NT individuals, suggesting a potential trend of reduced renal function in prehypertensive individuals. Spite of microalbuminuria have not showed a statistically significant difference between the groups, it is interesting to note that the PH group showed greater loss of urinary albumin than the NT group, a fact previously observed by Tenekecioglu et al. Thus, the urinary albumin leakage may be a manifestation of generalized vascular lesion already present in prehypertensive individuals (41).

The 24-h ABPM comparison of PH and NT groups revealed significant differences in SBP, DBP, MAP, cSBP, and cDBP, an underscoring the importance of early follow-up and, where necessary, pharmacological intervention to prevent HT onset (42). These differences, particularly in cSBP and cDBP, support prior studies linking higher central pressures with carotid intima-media thickness (43) and atherosclerotic plaque risk (44), as well as increased left ventricular mass (45) in prehypertensive individuals.

As expected, PWV was significantly higher in hypertensive individuals than in prehypertensive and normotensive individuals. PWV measured by Mobil-O-Graph is highly dependent on age and blood pressure, which were higher in hypertensive individuals in this sample, and this fact may have influenced this finding. Likewise, PWV in prehypertensive individuals was higher than in normotensive individuals, with a difference of 0.3 m/s, but this did not reach significance due to the sample size.

Moreover, differences in PP and PWV between PH and CHT groups across all periods (24-h, wakefulness, sleep) corroborate studies suggesting an acceleration of arterial stiffness from prehypertension to hypertension (46, 47). As PWV is a known predictor of cardiovascular events and mortality, its use in prehypertensive patients may aid in preventing sustained hypertension development (14).

The use of the Mobil-O-Graph® system for central blood pressure and arterial stiffness assessment was chosen due to its validated oscillometric method, which applies a transfer function to brachial waveforms to estimate central hemodynamic parameters. This technique offers a noninvasive, reproducible, and clinically applicable alternative to applanation tonometry, with demonstrated consistency across diverse populations. Furthermore, the decision to employ 24-h ABPM instead of the simpler triple office measurement was based on its superior ability to capture circadian blood pressure variations, detect masked or white-coat hypertension, and provide more reliable prognostic information. ABPM also enables the evaluation of nocturnal dipping and PWV under real-life conditions, which are critical for understanding early vascular changes in prehypertensive individuals.

### 4.1 Limitations

Although sample size calculation and *post hoc* power analysis confirmed adequate statistical power for the primary comparisons, subgroup analyses—particularly those involving age, sex, and cardiovascular risk stratification—may have been limited by the relatively small number of participants in the prehypertensive group.

Additionally, the cross-sectional nature of the study necessitates confirmation through longitudinal studies, particularly to identify predictive variables obtained via ABPM. Another important limitation is the method used to evaluate central hemodynamic parameters. This research employed a 24-h monitoring device, whereas many published studies have utilized applanation tonometry (46, 47).

The interpretation of PWV and central blood pressure values may be influenced by age, sex, and blood pressure levels. According to the Brazilian Guidelines on Hypertension (49), PWV should ideally be evaluated using reference percentiles stratified by age, sex, and cardiovascular risk factors. Due to limitations in sample size and the absence of standardized reference data across all strata, percentile-based adjustments were not applied in this study. This is recognized as a methodological limitation. Future research should incorporate stratified percentile-based analyses to improve the accuracy and clinical relevance of arterial stiffness assessments.

## 5 Conclusions

In summary, differences in ABPM parameters were observed across the groups during all three periods (24-h, wakefulness, and sleep) for both peripheral (SBP, DBP, MAP) and central (cSBP, cDBP, and PWV) hemodynamic variables. The PH group displayed higher peripheral and central pressure values compared to the NT group but lower values than the CHT group across all periods. This study demonstrates that prehypertensive patients present some biochemical-metabolic and in central hemodynamic parameters alterations, which may be suggestive of functional and structural alterations that predispose individuals to the development of hypertension.

## Data Availability

The raw data supporting the conclusions of this article will be made available by the authors, without undue reservation.
